# Optimizing methods to estimate zooplankton concentration based on generalized patterns of patchiness inside ballast tanks and ballast water discharges

**DOI:** 10.1002/ece3.3498

**Published:** 2017-10-16

**Authors:** Sarah A. Bailey, Harshana Rajakaruna

**Affiliations:** ^1^ Great Lakes Laboratory for Fisheries and Aquatic Sciences Fisheries and Oceans Canada Burlington ON Canada

**Keywords:** ballast water management convention, compliance monitoring, heterogeneity, representative sampling, sampling methods, spatial distribution

## Abstract

Zooplankton populations are spatially heterogeneous in nature and inside ship ballast tanks. Sampling methods should take heterogeneity into account, particularly when estimating quantitative variables such as abundance or concentration. It is particularly important to generate unbiased estimates of zooplankton concentration in ballast water when assessing compliance with new international ballast water discharge standards. We measured spatial heterogeneity of zooplankton within ballast water using three sampling methodologies. In‐tank pump samples were collected at fixed depths within the vertical part of the ballast tank (side tank). Vertical net‐haul samples were collected from the upper portion of the tank as a depth‐integrated and historically relevant method. In‐line, time‐integrated samples were collected during ballast discharge by an isokinetic sample probe, likely representing the double bottom part of the ballast tank. The bias and precision associated with each sampling method were evaluated in reference to the estimated average abundance of the entire ballast tank, which was modeled from the data collected by all methods. In‐tank pump samples provided robust evidence for vertical stratification of zooplankton concentration in the side tank. A consistent trend was also observed for in‐line discharge samples, with zooplankton concentration decreasing through time as the ballast tank is being discharged. Sample representativeness, as compared to the tank average, varied depending on the depth or tank volume discharged. In‐line discharge samples provided the least biased and most precise estimate of average tank abundance (having lowest mean squared error) when collected during the time frame of 20%–60% of the tank volume being discharged. Results were consistent across five trips despite differences in ballast water source, season, and age.

## 1 INTRODUCTION

Ballast water, utilized by large ships for stability and maneuverability, has long been recognized as a potent vector for the introduction of harmful aquatic organisms and pathogens. As some introductions can have irreversible negative ecological, economic, and human‐health impacts, there has been a coordinated international effort to reduce the risks associated with ballast water discharge. The recent ratification of the International Convention for the Control and Management of Ships’ Ballast Water and Sediments (International Maritime Organization 2004) will motivate many shipping companies to install ballast water treatment technologies, such as filtration and chlorination. Regulation D‐2 of the Convention stipulates a Ballast Water Performance Standard, limiting the number of viable organisms that can be discharged in ballast water; with respect to zooplankton (organisms ≥50 μm in minimum dimension), ships shall discharge <10 viable organisms per cubic meter. Related Guidelines for Ballast Water Sampling (International Maritime Organization 2008) state that sampling protocols to monitor compliance with Regulation D‐2 “should result in samples that are representative of the whole discharge of ballast water from any single tank or any combination of tanks being discharged.” However, collecting a representative sample poses a daunting challenge due to the large volumes of water carried by ships and limited access to ballast water tanks.

Spatial heterogeneity of plankton is a well‐documented phenomenon in nature resulting from large‐scale physical processes and small‐scale biological processes; at the scale relevant to ballast tanks (mm to tens of m), individual behaviors such as diel vertical migration, predator avoidance, and seeking food and mates are the dominant drivers of spatial distribution (Folt & Burns, 1999). Out of 76 primary publications on the biology of ballast water, only one study has explicitly examined the spatial distribution of zooplankton inside ballast tanks. Murphy, Ritz, and Hewitt (2002) sampled ballast water from three depths (0.5, 2, and 6 m) of two wing tanks during two trips using a diaphragm pump for enumeration and identification of common taxa. Mixed results were observed, in that there was no evidence for any trend by depth for bivalve larvae while crab zoea were up to 12‐fold more abundant in surface samples (Murphy et al., 2002); however, samples were not collected spanning the entire 13 m depth of the ballast tanks and abundances were typically low (limiting power to detect differences). The authors suggested that bottom‐to‐surface net hauls may generate more representative measurements of whole‐tank plankton concentrations than samples taken from a limited depth range (Murphy et al., 2002). However, as both vertical and horizontal structure of ballast tanks can affect distribution of ballast volume, adjustments may be required to estimate average zooplankton concentration across an entire ballast tank even if bottom‐to‐surface net hauls are conducted. Failure to account for error due to tank structure and plankton patchiness could result in poor estimates of zooplankton concentration, possibly leading to incorrect decisions during enforcement of the Convention.

As Regulation D‐2 applies to ballast water discharge, the Guidelines for Ballast Water Sampling recommend collecting samples from the ship's ballast piping during discharge, as close as practicable to the point of discharge, using an isokinetic sampling probe (International Maritime Organization 2008). There has been extensive research to develop equipment for collection of in‐line samples (e.g., Richard, Grant, & Lemieux, 2008; Wier et al., 2015) and to establish minimum required sample volumes (e.g., Frazier, Miller, Lee, & Reusser, 2013; Hernandez, Johansson, Xiao, Lewis, & MacIsaac, 2017; Miller et al., 2011), as well as multiple studies examining the distribution of zooplankton within sequential samples collected by in‐line sampling (e.g., Carney et al., 2013; First et al., 2013; Gollasch & David, 2013). In general, these studies report that plankton concentration can vary widely depending on the timing (sequence) of sample collection, leading to recommendations for collecting multiple 1‐m^3^ volume samples.

This study builds on earlier research to examine the representativeness of in‐tank and in‐line samples in relation to the whole‐tank population. The aim was to examine spatial heterogeneity of zooplankton within ballast water using three sampling methods, to model and estimate the average concentration of zooplankton across the entire ballast tank, and to determine the contexts under which different sampling methods are most representative (i.e., yield the best estimate of the tank average) in order to support assessments of ballast water compliance with Regulation D‐2 of the Convention.

## 2 MATERIALS AND METHODS

### 2.1 Survey and experimental methods

We measured zooplankton patchiness in a single ballast tank of the 216.6 m gearless bulk carrier M/V TIM S. DOOL, immediately before and during five separate ballast discharges at Clarkson, Ontario (Lake Ontario), between August 2015 and September 2016. The DOOL has six pairs of ballast tanks (port and starboard), each having connected double bottom and side tanks; the double bottom represents approximately half of the tank volume (i.e., 50% tank volume is realized when filled to 1.2 m depth). With the centerline ballast valve closed, port and starboard ballast systems operate independently, with separate ballast pumps (25,000 GPM) and sea chests. A sampling port (i.e., a flanged opening into the main ballast line) is present for collection of time‐integrated ballast water discharge samples on the starboard‐side discharge line owing to previous scientific research (Cangelosi, Schwerdt, Mangan, Mays, & Prihoda, 2011). The #3 starboard‐side ballast tank (830.6‐m^3^volume) was selected for study as the #3 tanks are first to be discharged during cargo loading. All tests were conducted on natural (untreated) ballast water held in tanks 19 hr to 7.5 days before discharge (Table 1).

**Table 1:** Detailed information describing the date and times of sample collection, source location, age, and depth of the ballast water for each ship trip

Trip	Date of sample collection	In‐tank stop time	In‐line start time	Ballast source	Ballast age	Ballast depth
1	26–27 August 2015	23:55	08:50	Hamilton, ON	3.3 days	8.20 m
2	22 September 2015	18:15	23:00	Montreal, QC	7.2 days	8.16 m
3	14 October 2015	11:55	13:00	Hamilton, ON	3.5 days	8.15 m
4	14 June 2016	06:00	07:00	Hamilton, ON	19 hr	8.01 m
5	24–25 September 2016	21:45	02:30	Hamilton, ON	2.6 days	7.36 m

**Figure 1:**
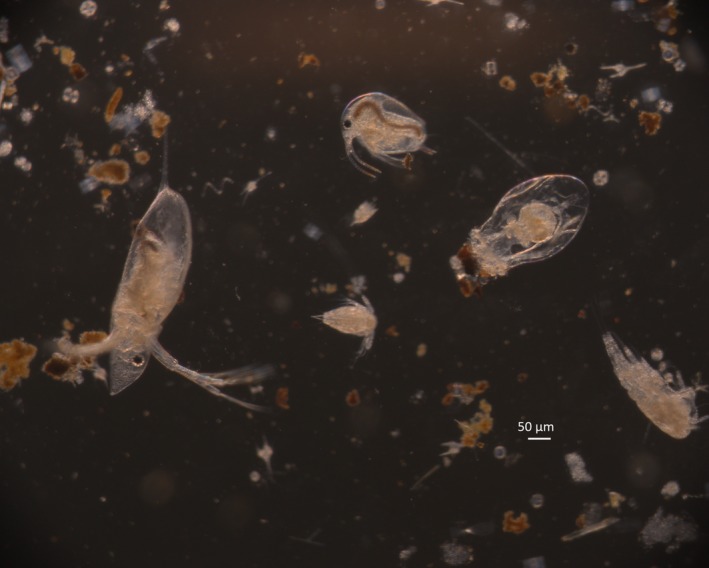
Photomicrograph of organisms ≥50 μm in minimum dimension, typically zooplankton, such as Cladocera, Copepoda, and Rotifera present in ballast water collected during trip 3 sourced from Hamilton, Lake Ontario

**Figure 2:**
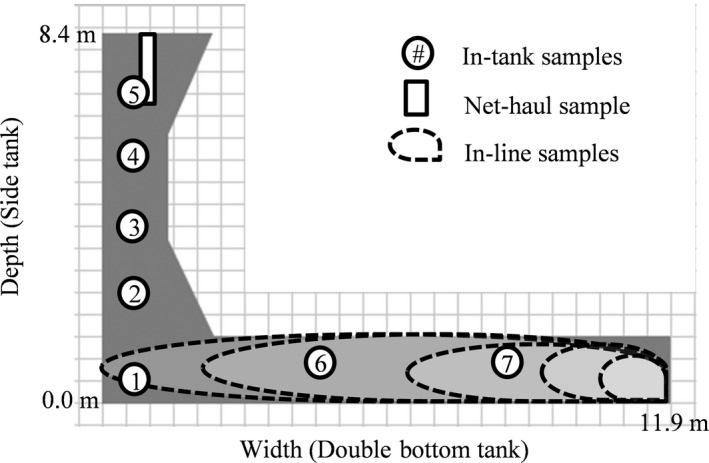
Cross‐sectional diagram of studied ballast tank (view from forward, in gray) showing the location and estimated coverage of the three sampling methods (drawn to scale). Sample points 1 through 5 were used for first four trips; sample points 1, 3, 5–7 were used for final trip. Spatial extent of in‐line samples is estimated assuming little tank mixing

Following the largest size category in regulation D‐2, this study focused on organisms ≥50 μm in minimum dimension, typically including taxa such as Cladocera, Copepoda, and Rotifera (Figure 1). Three sampling methods were used to measure spatial distribution of zooplankton (>50 μm in minimum dimension) in the side and double bottom parts of the tank (Figure 2). In‐tank, fixed‐depth samples were collected through the forward manhole access using a pump (Jabsco 777 Nitrile Impeller) and tubing (1’’ braided PVC) installed in the ballast tank. The fixed sampling points facilitated standardized sampling across experiments although water depth in the tank varied across trips. For the first four trips, pump samples were collected at five depths within the side tank (0.5, 2.5, 4.0, 5.6, and 7.1 m above tank bottom); for the fifth trip, two sampling points were relocated from the side tank to the double bottom to increase spatial overlap between in‐tank and in‐line sampling methods (Figure 2). Approximately 200 L was collected via each sampling point; a magnetic flowmeter (Seametrics WMP104‐100) measured sample volume prior to filtration through a semi‐submerged plankton net (35‐μm mesh). Each sample was condensed to 1 L volume for analysis, with waste water (filtrate) deposited into the adjacent ballast tank.

Vertical net‐haul samples were collected through the aft manhole access, but were restricted to the depth of the horizontal stringer plate inside the ballast tank. Ten to twelve vertical net hauls were conducted (depending on depth of the water) using a 30‐cm‐diameter, 1‐m‐long conical plankton net (35‐μm mesh) to achieve a composite sample of approximately 1,000 L. The contents of the net cod‐end were condensed to 1 L volume for analysis. Both pump and net‐haul samples were collected within 2 hr of the ship's arrival to port.

**Figure 3:**
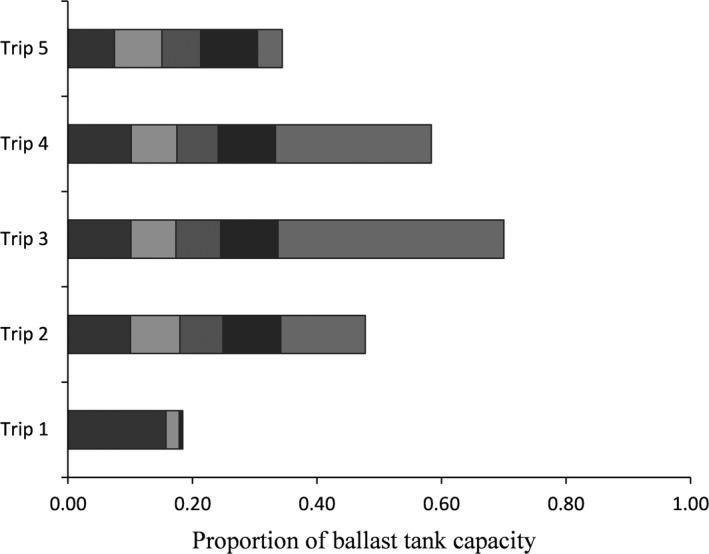
Proportion of total tank capacity discharged during collection of five continuous in‐line samples (indicated by different shading) by trip, illustrating variability due to fluctuations in ballast volume discharged by the ship. Note that the three terminal samples during trip one each represented <1% of tank volume

In‐line, time‐integrated samples were collected during ballast water discharge using a 2.54‐cm isokinetic sample probe and sample collection system similar to that recommended by Cangelosi et al. (2011). A magnetic flowmeter (Seametrics WMP104‐100) measured sample volume prior to filtration through a semi‐submerged plankton net (35‐μm mesh). Ballast water was discharged by gravity during trial #1, while the ship's ballast pump was used for later trials; the volume of ballast water discharged varied according to the demands of cargo operations. We used a General Electric TransPort PT878 Portable Ultrasonic liquid flow meter with clamp‐on transducers to measure ballast water flow rate and volume in the ship's piping upstream of our sample collection system; we adjusted our sample flow rate based on the measured flow rate in the ship's ballast piping to maintain a sampling rate lower than the calculated isokinetic sample flow rate. Samples were collected throughout discharge until the ballast pump lost suction/slowed (generally about 35 min after start of ballast discharge). The sample was diverted into a clean plankton net roughly every 7 min, resulting in five consecutive, continuous samples having, on average, 150 L in volume. The contents of each plankton net cod‐end were condensed to a 1 L volume for analysis. The ship's ballast tank gauges were monitored and the depth of water remaining in the tank noted each time the water was diverted to a new plankton net. While the in‐line sample collection was standardized in terms of subisokinetic sample rate, sample volume, and duration, there were differences in the proportion (volume) of ballast water discharged through the main ballast pipe during sample collection, ranging from <20% to >75% of the tank capacity (Figure 3). The difference in the proportion of ballast water discharged was particularly evident during trial one when ballast water was discharged by gravity.

All samples were immediately transported to the ship's wheelhouse for enumeration of viable zooplankton by microscopy. A 1‐ml subsample was taken from each 1 L sample after mixing by gentle inversion (5×) using a pipette with 3‐mm‐diameter tip opening; the subsample was placed into a modified Bogorov chamber (having five separate 1‐ml channels) for analysis under a Nikon SMZ800N dissecting microscope at minimum 30× magnification. Standard movement/response‐to‐stimuli techniques were used to enumerate viable, fully intact individuals ≥50 μm in minimum dimension. When zooplankton concentration was very low, multiple 1‐ml subsamples were counted or samples were concentrated to achieve counts of at least 20 live individuals per datum (with the aim of obtaining sufficient power for statistical analysis). When zooplankton concentration was very high, samples were diluted prior to subsampling. The volumes analyzed were carefully recorded to standardize estimates of zooplankton concentration at the original scale (per cubic meter of ballast water). All analyses were completed within 3.5 hr of sample collection.

### 2.2 Modeling and analytical methods

Generalized linear mixed effects models (GLMEM) were used for separate analyses of the data (counts per cubic meter) collected by in‐tank pump samples (examining any trend by depth) and in‐line discharge samples (examining any trend according to tank volume discharged) both within and across the five trips. Upon finding trends in zooplankton concentration for in‐tank samples by depth and in‐line samples by volume discharged, and noting neither in‐tank nor in‐line samples provided full coverage of the entire tank structure, it was determined that a whole‐tank average would be best estimated by combining estimates generated by all sampling methods. In‐tank and in‐line data were scaled to equivalent units statistically using a GLMEM (*fitglme* in Matlab^®^) with a best‐fitted polynomial function prior to pooling together and modeled using GLMEM with a polynomial function of depth/volume of discharge to estimate the average concentration of zooplankton across the whole tank. Finally, we compared the estimates generated by individual sampling methods to the tank averages. Note that we considered data collected by net haul most representative of the midpoint depth of the vertical haul, and data collected by in‐line samples most representative of the midpoint (in terms of the volume discharged) of the in‐line sampling sequence.

#### 2.2.1 In‐tank stratification and in‐line trend analyses

We assumed zooplankton concentration, *C*, across depth or volume discharged, was Gamma‐distributed, *C*[*j*] ~ Gamma (*k*, μ[*j*]), where *j* denotes random effects specified by ship trips, *k* is the shape parameter, and

(1) $$\mu \left[j\right]=E\left[C_{i}\left[j\right]\right]=\exp\left(\beta 1_{i}\left[j\right]+\beta 2_{i}\left[j\right]\cdot X_{i}\left[j\right]\right),$$

is the average (mean) concentration, allowing plankton patchiness along *X*_*i*_ [denoting the height from the tank bottom in in‐tank pump samples in the case i = 1, that is, *X*_1_ (m) and volume discharged for in‐line samples in the case i = 2, that is, *X*_2_ (m^3^)], where variance in concentration at any *X*_*i*_ is proportional to the square of the mean concentration at the given *X*_*i*_. Here, β1_*i*_[*j*] and β2_*i*_[*j*] are the coefficients of intercept and gradient, respectively, assuming random effects due to ship trips. Note that we used GLMEM, *fitglme* in Matlab^®^, with link function “Log,” assuming that errors associated with counts are exponential (multiplicative). The Gamma distribution gives the best continuous approximation to the discrete negative binomial probability distribution, especially when the count data are large (which also allows approximation to the normal distribution as a limiting case as per the central limit theorem).

#### 2.2.2 Estimate of average tank concentration

As in‐tank pump samples were mostly collected from the vertical side tank, having units of height, and in‐line samples were collected during the discharge of, on average, 50% of the tank (i.e., double bottom tank) having units of volume, we combined the sample estimates statistically to determine measures of volume by measures of height for the whole tank using the model:

(2) $$X_{1}=a{\left(\frac{X_{2}}{V}\right)}^{\;\;b}=W,$$

where *a* and *b* are scaling parameters; *V* is the total volume of the tank = 831 m^3^; *X*_1_ is the height (in meters) from tank bottom; and *X*_2_ is the volume discharged (in cubic meters) by the midpoint of the in‐line sample. Subsequently, the distribution of concentration (*C* ^1/2^, square‐root transformed) with respect to the standardized scale *W* (m or m^3^, depending on the conversion to *X*_1_ or *X*_2_, respectively) was modeled by a quadratic function:

(3) $$\text{sqrt}\left(C\left[j\right]\right)=\gamma 1\left[j\right]\cdot W{}^{2}\left[j\right]+\gamma 2\left[j\right]\cdot W\left[j\right]+\gamma 3\left[j\right],$$

allowing plankton concentration to nonlinearly decrease from the first in‐line sample to the last in‐line sample, and then nonlinearly increase from the deepest in‐tank pump sample to the uppermost in‐tank pump sample. Here, *j* denotes the grouping by ship trips. The scaling parameters *a* and *b* were estimated by minimizing the sum of squared errors (using Matlab^®^ *fminsearch*) of the square‐root‐transformed concentration data, *C* ^1/2^, with respect to models (Equations (2) and (3)) simultaneously, allowing parameters γ1, γ2, and γ3 to have group effects due to individual trips, while parameters *a* and *b* were fixed across all experiments. The square‐root transformation was used to stabilize the variance.

As concentrations observed near the tank surface estimated by net‐haul samples were consistently lower than that expected from the quadratic trends best‐fitted to in‐tank pump and in‐line sample data, as above, we modeled the trend in zooplankton concentration by height or volume discharged, *W*, given the data of all three sampling methods, using a third‐degree polynomial function, as:

(4) $$C\left[j\right]=\varphi 1\left[j\right]\cdot W{}^{3}\left[j\right]+\varphi 2\left[j\right]\cdot W{}^{2}\left[j\right]+\varphi 3\left[j\right]\cdot W\left[j\right]+\varphi 4\left[j\right],$$

to get the most accurate concentration estimates by height, *X*_1_, or volume discharged, *X*_2_, where *j* denotes the random effect due to ship trips. We estimated the parameters using GLMEM (*fitglme* in Matlab^®^), assuming data were Gamma‐distributed, allowing them to be overdispersed (i.e., to have patchiness). We used the “Log” link function, assuming multiplicative errors associated with the mean concentration given at any *W* in Equation (4). Thus, it gave *E*[*C*[*j*]] = exp[φ1[*j*]·*W* ^3^[*j*] + φ 2[*j*]·*W* ^2^[*j*] + φ 3[*j*]·*W*[j] + φ4[*j*]], yielding trends in the concentration data for each different trip, and also across trips as a general phenomenon, assuming random effects on coefficients due to trips. We also fitted generalized linear models to individual ship trip data separately assuming Gamma‐distributed concentrations and Log link functions.

Finally, we examined the errors in concentration estimates by particular samples by *W*, with respect to the estimated average concentrations given by the model (Equation (4)) by W for each individual trip. We standardized these errors across trips by dividing them by the average concentration of the whole tank, estimated for each individual trip, which was computed accounting for the tank's volume structure. We used the above standardized errors to estimate a standardized bias, variance, and mean squared error (MSE: bias^2^ + variance) in order to assess which samples, by height or by volume discharged, give the most accurate (i.e., least biased and most precise) estimate of the average tank concentration.

#### 2.2.3 Comparison of sampling methods

We tested whether the zooplankton concentrations estimated by the three sampling methods, regardless of the attributes of tank height or volume discharged, were significantly different from each other. Plankton concentration data, *C*, were assumed to be Gamma‐distributed, C[*i*] ~ Gamma (*k*, μ[*j*]), where *j* denotes random effects specified by ship trips, *k* is the shape parameter, and mean concentration μ[*j*] = *E*[*C*[*j*]] = exp(α1[*j*] + α2[*j*]·*M*[*j*]), allowing overdispersion (i.e., patchiness) of concentration as in previous models. Here, *M* denotes the method as a categorical dummy variable, taking values “0” and “1” and coefficients α1 and α2, having random effects due to trip. We used GLMEM, *fitglme* in Matlab^®^, with link function as “Log,” assuming errors are exponential (multiplicative). This allows the coefficient α2, which is the difference in average concentrations between the methods, to have random effects due to ship trips (caused by differences in zooplankton community composition) around a fixed generalized value of the same between methods across all surveys. This model specifically tests whether the difference in the estimates of average concentrations between the methods is “systematic,” that is whether α2 > 0 or <0 significantly regardless of the trip or as a general phenomenon, yielding relative biases between the methods.

## 3 RESULTS

**Figure 4:**
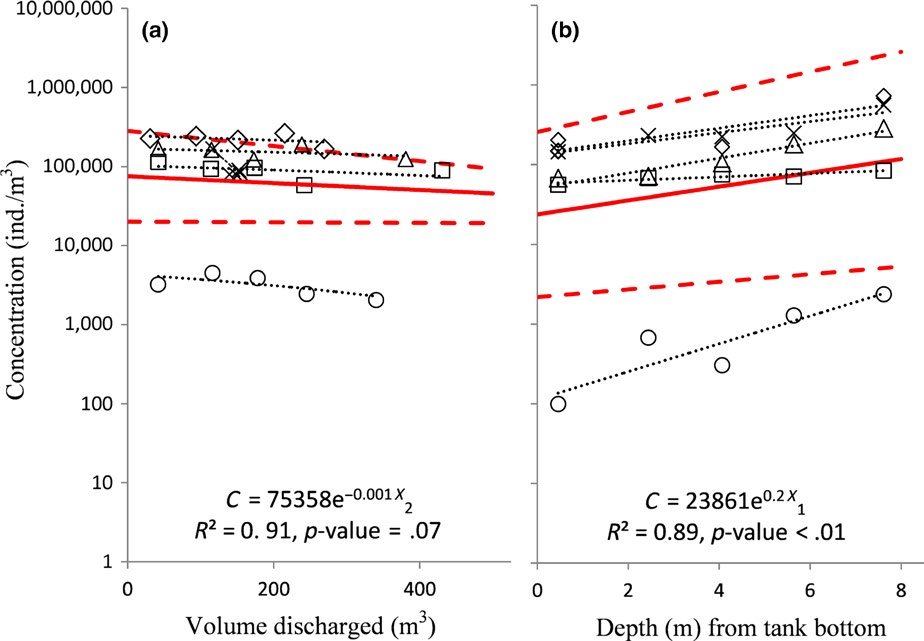
Point estimates of zooplankton concentration collected by (a) in‐line discharge samples and (b) in‐tank pump samples across five trips. Black dotted lines denote trends within trips, and solid red lines denote the generalized trend across trips, with dotted red lines indicating 95% confidence intervals

In‐line discharge samples showed a generalized trend in zooplankton concentration by volume discharged, *X*_2_, with initial discharge samples tending to have higher concentration than those toward nearly half of the tank volume being discharged (*R* ^2^ = 0.91, *p* = .07; GLMEM with Gamma‐distributed concentrations and link function “Log”; Figure 4a). While the statistical significance test suggests that there is a 7% probability of type 1 error, 91% of variation in the data is explained by the model. The low p‐value suggests that evidence for a trend in densities along the volume discharged across ship trips is fairly large, as a general phenomenon, with random effects on coefficients due to trip, and high R^2^ suggests high predictability of the model. Also note that the same model, assuming that the slope of the concentration with respect to the volume discharged (Figure 4a) is constant (fixed), that is, same for all trips, but only the intercepts have random effects due to ship trips, yields *p* = .05.

In‐tank pump samples also showed a significant trend (vertical stratification) in zooplankton concentration by height, *X*_1_, with increasing concentration with height above the tank bottom within the side tank (*R* ^2^ = 0.89, *p* < .01; GLMEM with Gamma‐distributed concentrations and link function “Log”; Figure 4b). The vertical trend across the trips indicates a general phenomenon, with random variation in coefficients due to differences across trips, with 89% of the variation in the data explained by the model. We get a similarly significant result for the case where it is assumed that the slope of the concentration with respect to the height (Figure 4b) is constant (fixed) for all trips, but only the intercepts have random effects due to ship trips.

**Figure 5:**
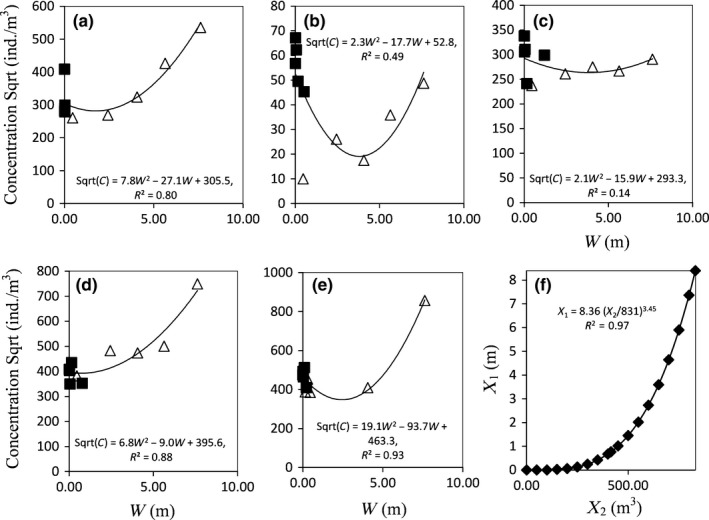
Panels (a–e): Quadratic functions (model Equation (3)) fitted to zooplankton concentration (square‐root transformed) data of in‐line and pump samples of each individual ship trip, given when parameterizing *a* and *b* in Equation (2), in combination with Equation (3). Squares = in‐line; triangles = pump. Panel (f): The resulting scaling model (Equation (2)), showing the relationship between height (*X*_1_) and volume discharged (*X*_2_) used to combine in‐tank and in‐line concentration estimates

The simultaneous calibration of the scaling model (Equation (2)) with the quadratic model (Equation (3)) effectively facilitated conversion of the unit of zooplankton counts between *X*_1_ (height in meters) and *X*_2_ (volume discharged in cubic meters), with the calibrated scaling model *W* = *X*_1_ = *a*(*X*_2_/*V*)^*b*^, where *V* = 831 m^3^ yielding *a* = 8.36 and *b* = 3.45 (Figure 5f, R^2^ = 0.97). The simultaneous quadratic functions fit well to the data of individual trips with respect to the standardized unit (*W*), except for trip number three (due to flatness), having *R* ^2^ = 0.80, 0.49, 0.14, 0.88, 0.93 and *p*‐values = .004, .09, .580, .001, <.001, by trip, respectively (Figure 5a–e). The fit of the generalized quadratic function to the data across all trips, after scaling to the standardized unit and considering random effects due to individual trips, yielded *p* < .05 with *R* ^2^ = 0.96.

**Figure 6:**
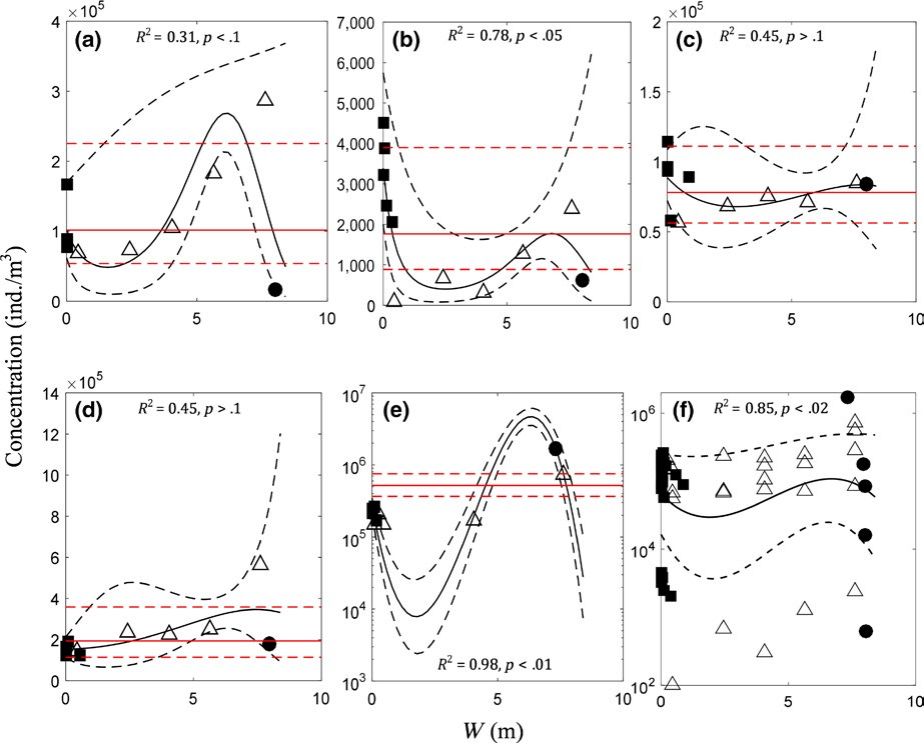
Estimate of whole‐tank zooplankton concentration as a third‐degree polynomial function of W = *X*_1_ = 8.36·(*X*_2_/831)^3.45^, by each trip in consecutive order (trips 1–5 in panels a–e), assuming Gamma‐distributed concentrations with Log link function in a generalized linear modeling framework. Panel (f): Generalized trend in distribution of zooplankton concentration across all trips estimated by GLMEM considering coefficients have random effects due to individual trips. In all panels, solid black lines show estimated mean trend with 95% confidence intervals (dashed black lines) and solid red lines show respective tank average with 95% confidence intervals (dashed red lines). Squares = in‐line; triangles = pump; and circles = net haul

The zooplankton concentration estimates from net‐haul samples were typically much lower than that estimated by the uppermost in‐tank pump samples. The uppermost in‐tank pump sample estimates did not fit the quadratic functions well (*p* > .1 and *R* ^2^ = 0.74); (Figure 5a–e). The third‐degree polynomial model (Equation (4)) used to fit to the data across all sampling methods of individual trips (Figure 6a–e) yielded *R* ^2^ values = 0.31, 0.78, 0.45, 0.45, 0.98 and *p*‐values <.1, <.05, >.1, >.1, <.01, by trip, respectively. While the fit was weaker for individual trips 1, 3, and 4, and significance was lower for trips 3 and 4 due to flatness of the curves, the generalized model fitted across trips yielded *R* ^2^ = 0.85 and *p*‐value < .017, strongly suggesting, both in terms of significance and predictability, that the pattern given by the third‐degree polynomial model is a general phenomenon (Figure 6f).

**Figure 7:**
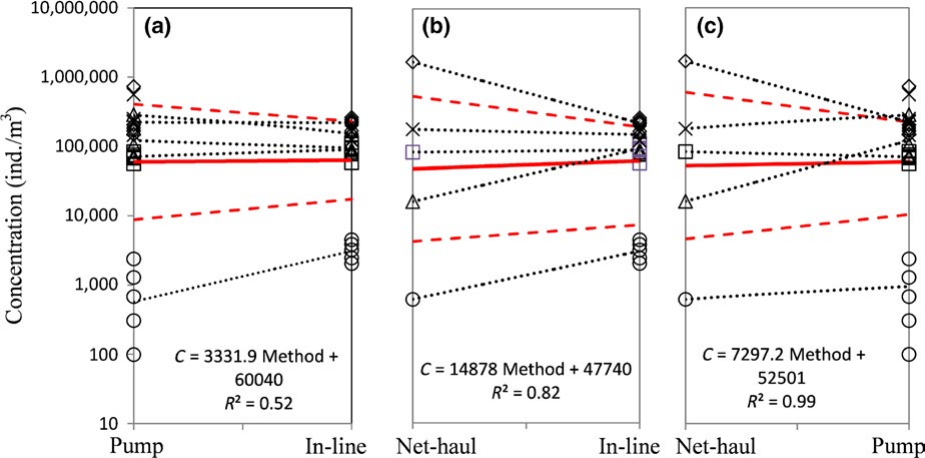
Comparison of zooplankton concentrations estimated by different sampling methods (a) pump versus in‐line; (b) net haul versus in‐line; and (c) net haul vs. pump, without accounting for the depth (pump), or the time sequence (in‐line). The generalized average concentration across trips estimated by GLMEM is denoted by solid red lines with 95% confidence intervals (dashed red lines); *p*‐values are .86, .67, .81, respectively. Trip‐specific differences in average concentrations between methods are shown by dashed black lines. Different symbols indicate individual trips

Figure 7 shows that there is no significant difference between estimates of concentration from net‐haul, in‐line, or pump samples if taken at random, that is, if we disregarded the depth (for in‐tank) or the point of volume discharged (for in‐line) of the sample. As there is a trend by depth and volume discharged, investigating the bias and the variance (precision) in sample concentrations by where/when they are taken (in terms of depth or volume discharged) with respect to the methods is critical for targeting a more accurate estimate.

**Figure 8:**
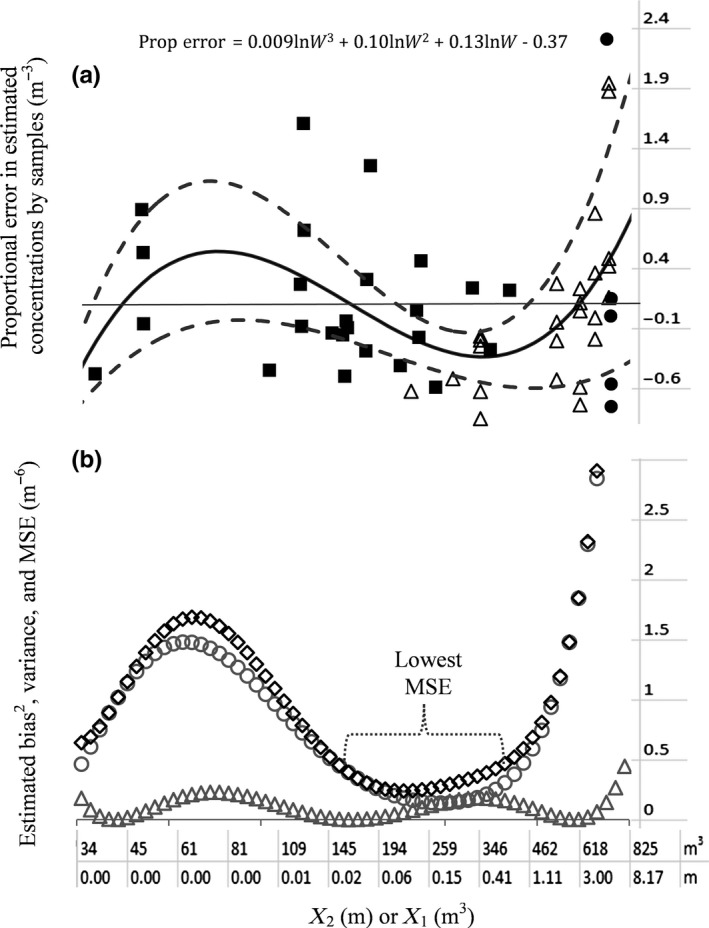
Panel (a): Bias (m^−3^) of the concentration estimates (solid black line) proportional to the respective tank averages of the individual trips, along *X*_1_ = 8.36.(*X*_2_/831)^3.45^, where *X*_1_ is height from tank bottom, and *X*_2_ is volume discharged by the midpoint of in‐line samples. Dashed lines show 95% confidence intervals. *p*‐Value <.02 for all coefficients of the polynomial model (*F* = 3.64, *df* = 3,51). Filled squares = in‐line; triangles = pump; and filled circles = net haul. Panel (b): Gray triangles = bias^2^ (m^−6^); gray circles = error variance (m^−6^); Black diamonds = Mean squared error (*MSE*) = bias^2^ + error variance (m^−6^)

The average concentration of the tank, estimated by the calibrated models of respective individual trips (Figure 6a–e in solid red lines), accounting for tank volume structure, yielded the errors associated with each sample concentration. Normalized errors, dividing the error by the respective mean concentrations of the tanks of the respective trips, are given in Figure 8a, with respect to Ln(*W*) on the *x*‐axis (note that the units in the graphs are given after converting them to *X*_1_ or *X*_2_). The bias along the Ln(*W*), (Figure 8b), is given by the expected value of the third‐degree polynomial function fitted to the errors (Figure 8a), and the variance (Figure 8b) calculated by the 95% confidence intervals of the model fitted (Figure 8a). The resulting MSE is given in Figure 8b, indicating where the most accurate estimates can be taken with respect to *W*, or in turn with respect to *X*_1_ or *X*_2_. Figure 8 indicates that concentrations estimated by net haul are generally less accurate (having higher MSE) than the other sampling methods (irrespective of *X*_1_, depth, or *X*_2_, volume discharged). This is due to its positive mean bias plus high imprecision (variance), in general (Figure 8b). According to the same figure, pump samples tend to have the highest accuracy (or lowest MSE) toward 0.5 m from the tank bottom. In‐line discharge samples have highest accuracy (lowest MSE) between 20% and 60% of the tank volume being discharged. That is, the concentration estimated by a random sample in this range is more likely to be close to the tank average than any other estimate, as a general principle.

## 4 DISCUSSION

This research provides strong and robust evidence for vertical stratification of zooplankton in the ballast side tank, as well as a trend of decreasing concentration with a greater proportion of ballast volume discharged by in‐line sampling. These general trends indicate that the depth or timing (in terms of the point of volume discharge) at which the sample is collected is critical for obtaining a sample that is representative of the entire tank average with greater accuracy. As results were consistent across five trips despite differences in ballast water source, season, and age, preliminary recommendations can be made concerning sampling methodology for monitoring zooplankton concentration in ballast water.

Historically, net‐haul samples taken from the ship deck through the ballast tank manhole access have been the preferred method for sampling zooplankton due to ease of use, speed of sampling and because the collection of an integrated sample through the ballast tank water column was previously suggested as the best method to capture an array of taxa (e.g., Gollasch et al., 2003; Murphy et al., 2002). However, it is recognized that no single method will effectively sample all taxa (Sutton, Murphy, Martin, & Hewitt, 1998). For this research, net‐haul samples were collected only from the uppermost portion of the side tank due to structural restrictions, a limitation which has frequently been encountered across international and domestic ships in prior studies (e.g., Adebayo, Zhan, Bailey, & MacIsaac, 2014; Briski et al., 2013; Murphy et al., 2002). Despite this physical limitation, it is interesting to note that, in general, across all trips, the concentration estimates taken by net hauls were not significantly different than the average concentration estimated by conducting five in‐tank pump samples or five in‐line discharge samples. However, in comparison with the tank average, the net‐haul estimates showed the highest MSE among methods, indicating net hauls are the method most unlikely to generate an estimate close to the tank average for a random ship. As net‐haul estimates are positively biased, if used for compliance monitoring, a net‐haul estimate indicating that the discharge standard has been met can be considered as a conservative “pass,” while a failure to meet the standard would be uncertain. One possible explanation for the high variance observed for net‐haul samples could be the semiquantitative measurement of sample volume. Net‐haul sample volume is typically calculated as the volume of a cylinder according to the haul depth and the radius of the plankton net opening; however, it can be difficult to measure the depth of the net haul accurately and if the net mesh is very fine or the net is towed too quickly, filter efficiency can decrease due to clogging, turbulence and back pressure (e.g., Sameoto et al., 2000; Wetzel & Likens, 2000). While a flowmeter is often used by oceanographers and limnologists for more accurate measurement of the volume filtered through large plankton nets, they tend not to be used on the very small plankton nets used in ballast tanks due to concerns about obstructing/causing turbulence at the net opening.

Collection of samples from a specific depth(s) of the ballast tank using a submersible or nonsubmersible pump with connected tubing is another method that has historically been used to collect zooplankton from ballast water tanks (e.g., Gollasch et al., 2003; Murphy et al., 2002; Ruiz & Smith, 2005). It is typically easier/more accurate to determine the sample volume collected by pump in comparison with plankton nets as flow meters can be used without restricting sample efficiency or water pumped to the ship deck can be measured using graduated pails. One disadvantage of pump sampling, however, is that the volume of sample collected may be limited by pump capacity and/or time available, some pumps may damage organisms during sampling, and some organisms may avoid the currents created in the water during sampling (Sutton et al., 1998). It also may not be possible to collect pump samples from all depths of a ballast tank without prior installation of fixed tubing (e.g., Hernandez et al., 2017), such that only the surface water of the ballast tank may be accessible for a typical compliance monitoring scenario. During our research, tubing was installed at multiple depths of the side tank and also extended into the double bottom for the final sampling trial. In general, we found samples collected about 2–3 m from the bottom (or less) give estimates closer to the tank average than net‐haul estimates, with zero bias and lower variance. The lowest MSE, or the highest accuracy, is achieved for pump samples collected from depths of about 0.1–1 m. For this reason, if used for compliance monitoring, the best depth for collection of a pump sample would be at the deepest part of the ballast tank (approximately 0.5 m off the bottom).

Collection of time‐integrated samples from a ship's ballast water discharge line is a relatively new method for ballast water sampling (e.g., Briski, Linley, Adams, & Bailey, 2014; Briski et al., 2015; Cangelosi et al., 2011). While this method requires much more equipment, labor, and technical expertise than historical methods, it is recommended for compliance monitoring as Regulation D‐2 is written as a discharge standard (i.e., the sample should be collected as close to the discharge point as is practicable). In addition, as many ballast water treatment systems apply a treatment process on discharge after the ballast water has drained from the ballast tank, a sample collected from the ballast water discharge line will be more representative than an in‐tank sampling method. While discharge line sampling logically is the best method, there are many practical limitations for its use, including inability to connect a sampling system to the discharge line due to absent, blanked or incompatible sample port flanges. Work is in progress to try to standardize the equipment associated with sample ports to make this process easier in the future (e.g., IMarEST 2016; ISO 2013). During our research, it was not possible to collect a time‐integrated sample for the entire duration of tank discharge due to the loss of suction and reduced flow rate of the ship's ballast water pump as the water level in the ballast tank was lowered. In our experience, a ship will normally begin to simultaneously discharge another full ballast tank or pump in water from the sea as ballast pump suction is lost to adjust for the loss in pressure as a tank is emptied. As our objective was to evaluate the representativeness of different sampling methods for a single ballast tank, we ceased sample collection prior to the introduction of additional water to avoid contamination of our samples and invalidation of our experimental design. We expect that the inability to collect time‐integrated samples for the entire duration of discharge of ballast water from a single tank will be a common phenomenon across ships, such that compliance monitoring will be limited to a similar volume as our experiments (i.e., no more than 75% of the tank volume). In‐line, time‐integrated sampling methods will produce estimates most representative of the tank average; however, due to variability in zooplankton concentration during discharge, the most accurate (or robust) estimate will be collected during the discharge of 20%–60% of the tank volume. Although the model indicates samples collected at the very beginning of discharge produce a fairly accurate estimate, the variability spikes immediately thereafter. Therefore, we would not recommend relying on estimates from samples collected at initial discharge; however, if used for compliance monitoring, an estimate based on sampling during initial discharge indicating that the discharge standard has been met can be considered as a conservative “pass,” as they are generally positively biased.

This study is the first to compare historical and newly recommended ballast water sampling methods for estimating the average concentration of zooplankton across an entire tank. As our results were consistent across trips despite differences in ballast water source, season, and age, we feel comfortable making preliminary recommendations for the collection of representative samples for compliance monitoring purposes. Additional research examining sample representativeness across different types of ballast tanks, different sizes of ships, and a broader selection of zooplankton communities (including those subjected to ballast water treatment) would be beneficial to confirm that the trends we observed are more generally applicable.

The International Convention for the Control and Management of Ships’ Ballast Water and Sediments will enter into force on September 8, 2017, with most ships expected to install ballast water treatment systems by 2020. While many countries have been requiring management of ballast water by open ocean exchange in recent years, compliance monitoring has typically been based on inspections of documents, crew interviews, and measurements of ballast water salinity. While the first two processes are likely to remain dominant means for monitoring compliance in the future, there will need to be significant development of protocols for representative sampling and analysis of ballast water should there be a need to proceed to biological assessment. Our results can be used to guide enforcement officers on methods and timing of sample collection. Theoretically, the best method to collect a sample that is representative of a whole ballast tank is to collect an in‐line, time‐integrated sample during the discharge of all water from the ballast tank; however, this is unlikely to be feasible for most compliance monitoring scenarios. Our results indicate that in‐line sampling for short durations during discharge of 20%–60% of the tank volume is expected to provide the most accurate, thus representative and robust estimates of the average concentration of zooplankton, at least for ballast tanks with connected side and double bottom tanks.

## AUTHOR CONTRIBUTIONS

SB conceived the project idea and the experimental design, conducted field work, and analyzed zooplankton samples. HR contributed to the experimental design, and conducted modeling and analyses of the data. Both authors contributed to the writing of the manuscript and gave final approval for the publication.

## CONFLICT OF INTEREST

None declared.
